# Correlation between Implant Surface Roughness and Implant Stability: A Systematic Review

**DOI:** 10.3390/dj12090276

**Published:** 2024-08-23

**Authors:** Marta Romero-Serrano, Manuel-María Romero-Ruiz, Mariano Herrero-Climent, Blanca Rios-Carrasco, Javier Gil-Mur

**Affiliations:** 1 Department of Periodontology, School of Dentistry, Universidad de Seville, C/Avicena S/N, 41009 Seville, Spain; romeromarta@uic.es (M.R.-S.); mmromero@infomed.es (M.-M.R.-R.); dr.herrero@herrerocliment.com (M.H.-C.); brios@us.es (B.R.-C.); 2 Porto Dental Institute, Periodontology Department, Symmetrya Prothesis, Av. de Montevideu 810, 4150-518 Porto, Portugal; 3 Bioengineering Institute of Technology, Faculty of Medicine and Health Sciences, Universidad International de Cataluña, C/Josep Trueta s/n, Sant Cugat del Vallés, 08195 Barcelona, Spain

**Keywords:** dental implants, surface, roughness, implant stability

## Abstract

The aim of this study was to find in the literature data on the relationship between implant surface roughness and implant stability achieved, from the time of placement to three months afterward, to help us to know what type of surface roughness is more favorable to guarantee implant stability and osseointegration. A systematic review was conducted in accordance with the PRISMA 2020 (Preferred Reporting Items for Systematic Review and Meta-analysis) statement, and the protocol was registered on the Open Science Framework. The specific inclusion and exclusion criteria were selected using the PICOS framework. The databases Medline (PubMed), Scopus, the Web of Science and The Cochrane Library were searched up to October 2023. The selection of studies and data extraction were conducted by two independent reviewers. The review included a total of 11 studies. A total of 1331 dental implant placements were identified. Two of the eleven selected studies were on humans in vivo, eight were on animals in vivo, and one was on animals in vitro. A statistically significant correlation between surface roughness and implant stability as measured by resonance frequency analysis (RFA) was not identified in ten of the eleven selected studies. It appears that there is no correlation between primary stability and the degree of implant roughness. However, there appears to be a correlation between the roughness of the implant and the degree of osseointegration, as indicated by bone-implant contact values. This correlation is more closely related to secondary stability. The great methodological variability makes it difficult to compare data and draw conclusions, so it would be desirable to agree on a common methodology to help draw appropriate conclusions from published studies.

## 1. Introduction

It is widely accepted that the surface texture of implants can influence the formation of bone at the implant-bone interface, with rougher surfaces generally resulting in enhanced osteointegration [1,2]. Over the past few decades, there has been a notable evolution in the surface modification of dental implants. Initially, implants were manufactured with highly polished surfaces. However, there has been a significant shift towards moderately rough surfaces, which are currently the predominant choice in the industry [3]. The objective of the implant surface modifications has been to create a more osteophilic surface, which accelerates the bone healing process and shortens the waiting time for implant loading [3,4]. The objective is also to attain optimal wettability, surface activity, or zeta potential in order to stimulate bone growth [5,6]. A favorable relationship was observed among surface texture, push-out strength, and bone-implant contact [7].

Various methodologies exist for creating a textured surface on the implant, including subtractive and additive techniques, such as mechanical, chemical, electrochemical, physical vapor deposition, and biological methods [8]. A number of studies have been published which report a variety of correlation levels between surface roughness and osseointegration, by comparing different types of implant surfaces [9,10,11,12,13,14,15].

There is now strong evidence that modification of implant surface roughness achieves clinical improvements such as increased survival, greater success in cases of immediate implants or early loading, or greater preservation of marginal bone [16,17,18,19,20]. indicating that alterations to microtopography can enhance surface area. This, in turn, has been linked to augmented levels of bone-to-implant contact (BIC), as observed for microtextured surfaces [21,22,23,24,25,26].

The measurement of the BIC value entails the removal of the implant, which results in the sacrifice of the animal. Consequently, researchers sought alternative methods for the assessment of osseointegration, aiming to achieve a more straightforward approach. They initiated the measurement of dental implant stability as a means of indirectly gauging the status of osseointegration [27,28]. The resonance frequency analysis (RFA) technique, utilizing the Osstell device (Osstell AB, Gothenburg, Sweden), has become a prominent methodology for evaluating implant stability in both clinical practice and animal research, as proposed by Meredith [29]. This method is founded on an evaluation of the oscillatory frequency of the implanted device in the surrounding bone tissue, as a consequence of a magnetic pulsing stimulus. This is then transformed into ISQ values (implant stability quotient), which fall within the range of 1 to 100 [29,30]. 

In considering the stability of the implant, two distinct phases must be taken into account. The term ‘primary stability’ is used to describe the absence of implant mobility when it is inserted into the bone [30]. Primary stability is a necessary condition for osseointegration to ensure that the implant is correctly anchored to the bone. Furthermore, it is essential for implant stability during the initial postoperative period, during which it gradually decreases due to bone remodeling. This decrease ultimately leads to the establishment of secondary stability, which is an inevitable consequence of the osseointegration process [31]. Secondary stability increases over time in accordance with the stiffness of the bone-implant interface, reaching its maximum level 5–6 weeks following implant implantation [32]. RFA has been demonstrated to be an effective method for monitoring the progression of implant osseointegration, as it provides a clinical assessment of the quality of the bone-implant interface during both the primary and secondary stability phases [33].

Adequate stability ensures that there is no detrimental micro-movement at the interface between the bone and the implant, allowing for proper healing. For successful osseointegration, micromovement between the implant and the surrounding bone should not exceed a threshold of 150 µm. Micromovement above this threshold can adversely affect bone remodeling and lead to fibrous tissue formation and implant failure [31,32,33,34].

Few systematic or narrative reviews in the literature have analyzed the relationship between surface roughness and dental implant stability. But we have found numerous recent studies in which different implant surfaces were compared trying to find the influence that surface exerts on the implant stability and osseointegration. In these papers we found a great variability of data, variables, protocols and methodologies, which sometimes makes it very difficult to compare results and draw conclusions that can be easily applied to daily practice. The aim of this study is to examine the correlation between implant surface roughness and stability achieved from the time of insertion up to three months post-operatively, covering all existing study models -human, animal, experimental-, and selecting articles that can be compared with each other, allowing us to extract data that will help us to know what type of surface roughness is more favorable to ensure implant stability and osseointegration.

## 2. Materials and Methods

The purpose of this systematic review was to evaluate the influence of surface roughness on the stability of dental implants, measured by resonance frequency analysis (RFA) and/or insertion torque (IT), in different study models, animal in vivo, human in vivo, and in vitro studies. Stability, as measured by ISQ scores, was the primary outcome. This review was performed in accordance with the PRISMA 2020 (Preferred Reporting Items for Systematic Reviews and Meta-Analyses) [35]. The review protocol is registered in the Open Science Framework archive link; https://archive.org/details/osf-registrations-qr43g-v1. Registration DOI; https://doi.org/10.17605/OSF.IO/QR43G (accessed on 14 July 2024).

### 2.1. Research Question

We used PICOS framework to pinpoint our research questions:

Population; studies researching the influence of dental implant surface on implant stability, in animal or human model.

Interventions: influence of implant surface on implant success, determined by RFA and IT. Comparison; studies comparing different surfaces of titanium implant, commercial and/or experimental surfaces. Outcomes; primary stability of dental implants. Study design; studies comparing different implant surfaces with or without a control group.

The PICOS question would be as follows; Which type of implant surface roughness will have the greatest influence on implant stability measured with RFA and/or IT, in human or animal or experimental models, when comparing different surface types? [36,37].

### 2.2. Inclusion and Exclusion Criteria

The specific inclusion and exclusion criteria were selected using the PICOS framework.

#### 2.2.1. Population

Inclusion criteria; studies that evaluated the effect of the modification of the surface topography on the stability of dental implants.
Exclusion criteria; studies that do not describe the roughness characteristics of the surfaces under study.

#### 2.2.2. Interventions

Inclusion criteria.

- Studies evaluating the influence of surface roughness on implant stability which could be measured with RFA and IT.
- The period of measurement began at the time of implant insertion and continued for a maximum of three months.
- Any animal or human study; in vivo or in vitro model.

Exclusion criteria.

- Studies with bone defects made artificially in the bone.
- Studies with any adjunctive therapy.
- In vitro studies that do not use animal tissues.
- Immediate load in vivo studies

#### 2.2.3. Comparison

Inclusion criteria; studies comparing the influence of surface roughness of different titanium dental implants with each other or with a control surface.
Exclusion criteria; studies with zirconio implants or implants with surface other than titanium.

#### 2.2.4. Outcomes

Inclusion criteria; studies evaluating implant stability measured with RFA (ISQ values) and/or IT.

Exclusion criteria.

- Studies that didn’t use RFA or IT to asess primary stability
- Studies using other methods of primary stability assesment

#### 2.2.5. Study Design

Inclusion criteria: research studies comparing different implant surfaces with or without a control group.

Exclusion criteria: case studies.

### 2.3. Search Strategy

An electronic search of the Medline (Pubmed), Scopus, the Web of Science and The Cochrane Library databases was conducted. A comprehensive search of the relevant databases was conducted up to October 2023. The references cited in the selected articles and in various systematic reviews were then subjected to a manual screening. They were considered only the publications written in English and no time restriction was applied. The research was concluded with a manual screening of the references cited in the various studies reviewed. 

In regard to the Medline (Pubmed) library, the following strategy was applied in the search: (“dental implants” [MeSH] OR dental implants [TIAB] OR “dental implant” [MeSH] OR dental implant [TIAB] OR dental implants [TIAB]) AND (“surface properties” [MeSH] OR surface properties [TIAB] OR properties, surface [TIAB] OR surface property [TIAB] OR Implant Surface [TIAB] OR Implant surface treatment [TIAB] OR in vivo study [TIAB] OR in vitro study [TIAB] OR animal study [TIAB]) AND (resonance frequency analysis [TIAB] OR primary stability [TIAB] OR implant stability [TIAB] OR insertion torque [TIAB]) AND (“Osseointegration” [MeSH] OR Osseointegration [TIAB]). 

The following strategy was employed for the Scopus database search: ((“dental implants” OR “dental implant”) AND (“surface properties” OR “implant surface” OR “implant surface treatment” OR “implant roughness” OR “roughness” OR “in vivo study” OR “in vitro study” OR “animal study”) AND (“resonance frequency analysis OR “primary stability” OR “implant stability” OR “insertion torque”) AND (“osseointegration”)).

### 2.4. Selection of Studies

Two trained reviewers (MRS and MMRR) conducted independent screening of titles and abstracts for potential inclusion of eligible papers, in accordance with the established inclusion criteria. Initially, publications deemed irrelevant, or duplicates were excluded based on the title. The abstracts of the remaining search reports were then examined, and finally, the full texts of all the remaining reports were assessed. Any discrepancies were resolved through discussion, reexamination of the relevant material, and consultation with the last author (JGM). Subsequently, the studies that were excluded and the rationale for their exclusion were documented. 

### 2.5. Data Extraction

Data extraction was conducted by two reviewers (MRS and MMRR) in an independent manner through the use of a predesigned extraction form. When feasible, some additional information was taken from the selected studies: names of the authors, publication year, sample size, research model, types of surfaces, roughness values, ISQ values, insertion torque values, evaluation times, and outcomes. Any inconsistencies or discrepancies were addressed through deliberation, a thorough review of the material in question, and a consensus, if necessary, or by consulting the last author (JGM).

### 2.6. Stability Values

All selected studies employed resonance frequency analysis (RFA) to ascertain implant stability. This technique assesses the stability in relation to the rigidity between the dental implant and the bone tissue system through the use of a magnetic peg connected to the implant. The values obtained, designated as ISQ values (implant stability quotient), span a range from 1 to 100.

### 2.7. Roughness Values

The chosen studies needed to include the commonly recognized scientific parameters for describing surface roughness, such as the 2-dimensional Ra (average profile roughness) and/or the 3-dimensional Sa (average area roughness).

We followed the classification for surface roughness for oral implant established by the consensus report of implant surfaces and design (working group 4) and specified by Wennerberg (2009) [1,10].

- Smooth surfaces: Sa value < 0.5 µm (e.g., polished abutment surface).
- Minimally rough surfaces: Sa value 0.5–1 µm (e.g., turned implants).
- Moderately rough surface; Sa value 1–2 µm (e.g., most commonly used types).
- Rough surfaces; Sa value > 2 µm (e.g., plasm-sprayed surfaces).

### 2.8. Publication Bias Analysis

In accordance with standard practice for meta-analysis, two researchers (MRS and MMRR) undertook an independent assessment of the risk of bias in all reports included in the review. In accordance with the criteria established by the Cochrane Collaboration in its Cochrane Handbook for Systematic Reviews of Interventions, the following elements were subjected to evaluation: 1. Random sequence generation; 2. Allocation concealment; 3. Patient blinding; 4. Outcome blinding; 5. Incomplete outcome data addressed. The included publications were grouped into the following categories: A. The study was deemed to have a low risk of bias if all of the criteria were met, indicating that potential bias did not significantly impact the results, B. The study was classified as having a high risk of bias if one or more criteria were not met, suggesting that possible bias may have a notable impact on the reliability of the results, C. The study was categorized as having an unclear risk of bias when very few details were available to classify it in the other two categories. (Table 1).

## 3. Results

### 3.1. Study Selection

**Figure 1:**
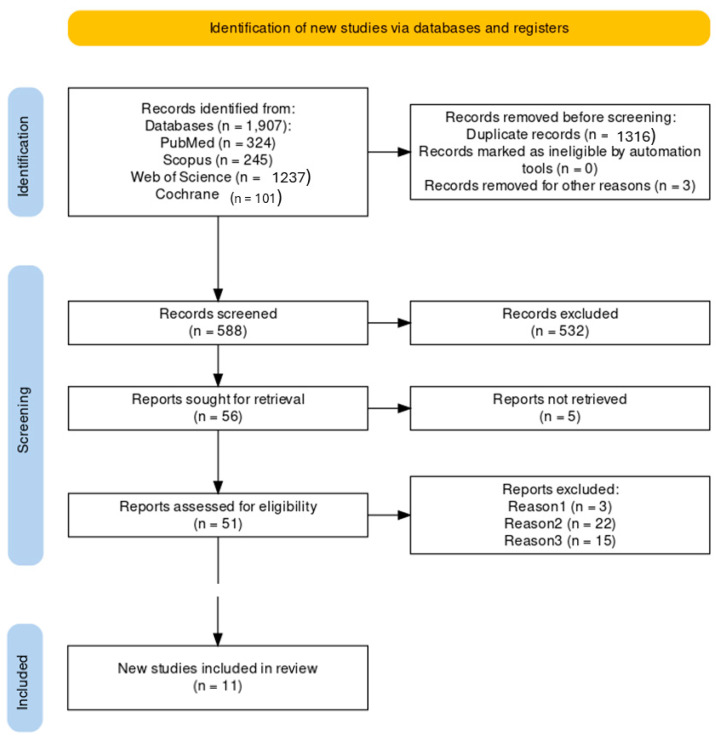
PRISMA flowchart showing the search strategy, screening and selection of the articles.

A PRISMA flowchart (Figure 1) provides a graphical representation of the process of literature search and study selection. The search in the different databases identified 1907 records as potentially relevant from the following database: PubMed (324), Scopus (245), Web of Science (1237), Cochrane (101). Before screening, we removed 1316 duplicated records, and 3 records for other reasons (n = 1319). Subsequently, 588 records were subjected to screening, resulting in the exclusion of 532 records deemed to be off-topic. A total of 56 reports were sought for retrieval; 5 of these were not retrieved, and 51 were assessed for eligibility. For various reasons, 40 reports were ultimately excluded from further consideration.

- Reason 1; In vitro studies but without using animal tissue. (n = 3)
- Reason 2; does not provide data on surface roughness values, or values are very similar and not comparable. (n = 22)
- Reason 3; does not use RFA or IT as stability measures (n = 15)

After the different selection phases, 11 studies were included in our review and selected for data extraction Refs. [38,39,40,41,42,43,44,45,46,47,48], Cohen’s kappa coefficient was utilized to assess the inter-rater agreement between the two reviewers. The Kappa value was 0.84.

### 3.2. Included Study Characteristics

The details of the included studies are provided in Table 2. The total number of dental implant placements was 1331. Two of the eleven selected studies were on humans in vivo [40,43], eight were on animals’ in vivo model [39,40,41,42,44,46,47,48], and one was in an in vitro animal model [45]. All studies evaluated the mean ISQ values. Insertion torque was also measured in three studies [38,40,45]. Histomorphometric analysis (bone to implant contact values) was performed in six studies [39,40,41,42,44,47].

The measurement intervals for ISQ evaluation in the included studies were different. ISQ was assessed at the time of implant placement (baseline) in two studies [38,45]. The rest of the articles showed a great variability in the moments of measurement of the implant stability by RFA, being different the number of measurements and the times in which the measurement takes place.

### 3.3. Risk of Bias Assessment

A single study included in the analysis was deemed to have a low risk of bias [39]. With regard to the remaining ten studies, our assessment was that their respective risks of bias were not clearly discernible. Refs. [39,41,42,43,44,45,46,47,48] were primarily attributable to selection bias. None of the studies included in this analysis were deemed to be at high risk of bias (Table 1).

### 3.4. Statistical Correlation

In ten of the eleven selected studies there was no statistically significant correlation between surface roughness and resonance frequency analysis [38,39,40,41,42,43,44,45,47,48]. Only in one study was there a statistical correlation [46]. Insertion torque was measured in three studies [38,40,45]. It should be noted that the studies included in the review did not assess ISQ and IT at both the implant insertion and post-osseointegration phases; in some cases, only one of these phases was considered.

Six papers [38,39,41,42,44,47] included histomorphometric studies, and in five of these, no correlation was observed between RFA-BIC [38,39,42,44,47]. A single study [39] did, however, identify a correlation between implant roughness and osseointegration, indicated by BIC values.

## 4. Discussion

The objective of this study was to undertake a comprehensive review of existing literature on the correlation between the degree of surface roughness of dental implants and the level of implant osseointegration achieved for a period of up to three months following the procedure of implantation. In a previous in vitro research study, we found that the surface roughness has no bearing on primary stability [45].

Adequate and substantial primary stability at the time of insertion is a crucial factor influencing the success of dental implants. The degree of achieved primary stability is influenced by a number of factors. Huang et al. [32] provide an exhaustive review of the literature, offering a comprehensive overview of the factors that have been identified as influencing ISQ measurements. From the 17 basic factors influencing ISQ measurements, they identify six that are predictive of clinical outcomes and can be rapidly assessed in practice. These are implant location, immediate versus delayed implantation, implant length and diameter, macro and micro design, bone quality, and implant surgery. So, stability depends not only on the characteristics of the implant surface but also on many other factors not considered in our review. So, this point could lead to a bias in the evaluation of the results extracted from the different studies selected. However, even if we take this into account, it seems to us very important in the light of the multiple articles published, to assess whether the surface roughness factor could be related to the ISQ value or to the insertion torque, and secondly with the histomorphometric studies of some of the included studies.

Different authors have reported on the benefits that a surface with a certain level of roughness brings to the primary stability and osseointegration of an implant at both the mechanical and cellular levels. Microroughness improves mechanical stability, and enhances BIC levels, thus maximizing the interlock between the mineralized bone and the implant surface [49,50,51]. For this reason, researchers have tried to find the most suitable degree of roughness to achieve a higher quality surface that guarantees a better and faster osseointegration. It can be seen, then, that primary stability during insertion and at the conclusion of the osseointegration process represents a critical factor in the success of dental implants. The biomechanical quality of osseointegrated bone has been demonstrated to be affected by the surface roughness of dental implants. In comparison with bone that has been embedded in machined surfaces, bone that has been embedded in rough-surfaced implants has been observed to be harder and stiffer [49,52]. However, the impact of implant microstructure on stability remains inconclusive; consequently, the aim of this study was to evaluate the effect of surface roughness on implant stability. 

Two of the selected studies measured implant stability only at the time of insertion [38,45]. The rest of the papers [39,40,41,42,43,44,46,47,48] measured stability also over the duration of the study, which varied according to the methodological design of the study, between one week and three months. These studies therefore measured both the primary stability achieved at the time of implant insertion and the biological or secondary stability achieved later during the process of osseointegration. Most studies reviewed have shown that there is no statistical correlation between implant surface roughness and implant stability as measured by resonance frequency analysis (RFA), neither in primary nor in secondary stability. These results coincide with those published by other authors [30,48]. Nevertheless, Souza et al. [46] discovered a correlation between surface roughness and RFA, comparing a machined surface with one that had undergone sandblasting and acid etching modification.

Only three articles measured primary stability using RFA and insertion torque (IT) at the time of insertion [53,54,55]. Dagher [39] found no statistical significance between the two values. In this sense, in a systematic review Lages [56] found no correlation between IT and primar stability, concluding that a high IT does not necessarily mean a high RFA. A number of factors can influence the correlation between torque and resonance frequency analysis, such as: surface treatment, implant shape or even variations in bone density [38,57].

Do Carmo [38] and Romero [45], found a positive correlation between RFA and IT, and conclude that the roughness of dental implants does not influence their primary stability values according to the IT and ISQ results. This can be explained by the hypothesis that surface roughness exerts its influence primarily on secondary stability, given that the formation of new bone on the implant would enhance its fixation and impede the implant’s movement within the surrounding bone. Primary stability would not depend on surface roughness but would be more sensitive to the insertion stress exerted by the clinician when placing the dental implant, because the dimensions of the hole created by the drills are smaller than the implant diameter. This results in a compressive effect between the implant surface and the surrounding bone, which facilitates primary stability. Additionally, the macrodesign of the implant exerts an influence on this stability. Excessive compression at the time of implant insertion can lead to bone necrosis. This is the reason why the clinician’s skill would be critical in achieving primary stability [40,43,45]. 

In the same direction would be the results of Sul et al. [47] who investigated implant stability by RFA of different implant surfaces. Baseline ISQ values at implant placement indicated no significant differences in primary stability among the implants. There was no correlation between surface roughness and RFA values. However, all implants showed high increases in stability at the end of the six weeks of the study, being statistically significant (secundary stability), so RFA values in this study were good reflections of the osseointegration process. Similar results were reported by Del Fabro [40], who found no statistical correlation between surface roughness and RFA, but his results support the hypothesis that may exist a correlation between the surface roughness and the osseointegration degree, being the highest bone-implant contact (BIC) values associated with the highest values of profile roughness at linear (Ra) and surface (Sa) level. This correlation between osseointegrantion and roughness surface have been reported in different studies, and would demonstrate according to these authors, how the response of bone is influenced by the surface topography [58,59]. 

This last conclusion does not coincide with the results of other authors such as Qamheya [44], Aparicio [60] or Rompen [61], who conclude that the measured RFA values are inconsistent with the consequent biomechanic and histomorphometric results. In the same way, Strnad [48] found no statistical correlation between RFA and BIC values, indicating that different BIC values may have similar stability values, or that implants with similar BIC values may have different ISQ values. All these contradictions among the published studies show the enormous difficulty in drawing clear conclusions that can be applicable to daily practice.

Although most of the studies reviewed find that the level of roughness does not influence implant stability, these studies do not always make clear the limits between the primary stability at the moment of placement and the secondary stability during the healing process of the implant. From the literature reviewed it is clear that in order to evaluate the true influence of the implant surface roughness, the most useful studies are those that evaluate the relationship between roughness values (Ra, Sa) and the secondary stability, as well as with the percentage of bone-implant contact, that is, that assess the degree of osseointegration (histomorphometric studies), although, as noted above, the results are contradictory.

The heterogeneity of the studies in terms of methodology represents a significant limitation of our review, making a direct comparison of results challenging. So, the selected articles presented different study models, different timing of evaluation of the variables, different types of surfaces to be compared, different variable to be measured, and different ways of measuring roughness values. Some authors claim that the reason for the difficulty in comparing these studies could lie in the varying quality of the surface assessment techniques and in the different definitions of surface roughness adopted in the papers [1,40]. It must also be acknowledged that the study is limited by the fact that new variables affecting bone formation in titanium dental implants have emerged, and thus require further investigation. One factor to consider is the residual compressive stress generated on the titanium surface as a result of the projection of abrasive particles, which provide roughness. The evidence suggests that elevated compressive stress promotes hydrophilicity and protein adsorption at the implant surface, which in turn stimulates enhanced bone formation. This hypothesis has been supported by several studies. Furthermore, additional aspects of the crystalline structure, including grain size and the orientation of hexagonal grains, result in the formation of textures that significantly alter the properties of titanium. It is evident that these latter factors exert a comparatively limited influence on osseointegration outcomes, yet they warrant consideration [45,62,63,64,65].

Furthermore, another limitation is associated with the potential for bias, which is also linked to the methodological shortcomings of the selected studies. Consequently, only one of the studies [40] was deemed to exhibit a low risk of bias. The remaining studies were deemed to exhibit an unclear risk of bias, predominantly due to concerns pertaining to selection bias, specifically with regard to sequence generation and allocation concealment. In this regard, the risk of bias of the studies included in the review was assessed using the Cochrane Collaboration tool, despite the fact that this tool has been designed for the assessment of the risk of bias in randomised controlled trials (RCTs). As the tool typically employed to quantify the risk of bias in non-randomised studies exhibits numerous similarities with the Cochrane tool, we elected to utilise the latter for all selected studies, irrespective of whether they constituted RCTs. This approach may, however, entail a potential limitation of the study. Our results support the hypothesis that there would be a correlation between the degree of surface roughness and the degree of osseointegration achieved, as assessed by the percentage of bone-to-implant contact. However, the correlation between the degree of roughness and implant stability measured with RFA did not show positive results.

It would be desirable that future studies try to unify methodological criteria to be able to compare their results and try to quantify the true influence of changes in implant roughness on the properties shown by the implants in the osseointegration process. It would also be interesting to be able to evaluate other possible advantages such as the influence of roughness on loading times, on its behavior in poor quality bone or its effects on the growth of different bacterial strains.

## 5. Conclusions

The findings of this review indicate that implant surface roughness does not affect primary stability; it is instead directly implicated in secondary stability. This is because surface texture affects the rate at which osseointegration occurs, thereby influencing the overall stability of the implant.

A correlation has been observed between the degree of surface roughness and the degree of osseointegration achieved, defined as bone-implant contact.

Significant methodological variability hinders the ability to compare data and draw meaningful conclusions, highlighting the necessity for a unified methodology to facilitate the interpretation of published studies.

## Figures and Tables

**Table 1:** Level of risk of bias on selected studies. Unclear risk: Yellow. Low risk: Green. High risk; Red. The five criteria assessed are as follows: 1. random sequence generation (selection bias); 2. allocation concealment (selection bias); 3. patient blinding (performance bias); 4. outcome blinding (detection bias); 5. incomplete outcome data addressed (attrition bias).

	Global	1	2	3	4	5
Do Carmo [38]	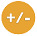	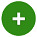	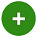	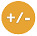	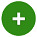	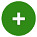
Dagher [39]	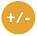	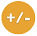	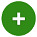	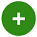	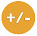	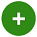
Fabbro [40]	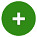	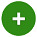	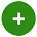	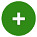	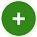	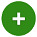
Gotlow [41]	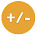	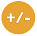	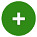	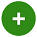	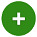	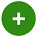
Kim [42]	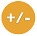	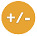	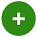	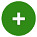	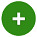	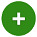
Körmöczi [43]	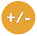	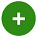	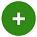	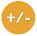	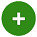	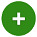
Qamheya [44]	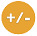	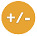	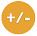	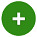	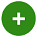	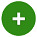
Romero [45]	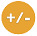	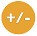	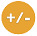	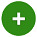	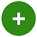	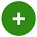
Souza [46]	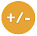	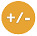	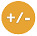	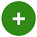	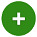	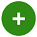
Sul [47]	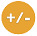	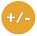	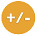	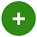	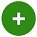	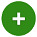
Strnad [48]	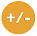	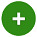	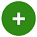	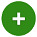	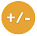	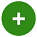

**Table 2:** Characteristics of the included studies.

Study	Sample Size	Study Type	Surface Treatment	Roughness Values	ISQ and TI Values	Evalution Time	Results Conclusions
Dagher (2014) [39]	32	Animal, In vivo. Prospective. 8 sheep	1. TiO_2_ blasted and etched (Euroteknika) 2. Anodized (Ti Unite) 3. Sandblasted (SLA) 4. Sandblasted and etched (SLActive, Straumann)	1. Euro-teknika: Moderately rough. Values not available 2. TiUnite: Sa 1,1-1,3 µm. SDR 37% 3.SLA 4. SLActive; Sa 1,5	ISQ * Baseline 1. 75.46 2. 78.28 3. 73.5 4. 73.5 IT 1. 84.4 2. 77.7 3. 74.8 4. 57.3	ISQ and IT Only at implant insertion	No significant correlation RFA-BIC. No correlation AFR-IT. No statistical correlation between roughness Surface-RFA.
Del Fabbro (2017) [40]	24	Animal, in vivo. Minipigs	1. Al_2_O_3_ blasted and etched (Dental tech) 2. Hydroxyapatite blasted and etched (Dental Tech) 3. Double etched (Politécnico) 4. Anodic spark deposition CPTi (Eurocoating) 5. Anodic spark deposition Ti (Eurocoating) 6. Blasted and etched -SLA type- (Nobil Bio Research)	1. Sa; 1.4 µm. Ra 1.6 µm. SDr 71% 2. Sa; 2 µm. Ra 2.1 µm. SDr 170% 3. Sa; 1.3 µm. Ra; 1.4 µm. SDr 66% 4. Sa; 1.1 µm. Ra 1.3 µm. SDr 54% 5. Sa; 1 µm. Ra 1.1 µm. SDr 39% 6. Sa; 1.5 µm. Ra 2.2 µm. SDr 88%	ISQ * Baseline 1. 66.5 2. 72.5 3. 69.3 4. 65.9 5. 69 6. 70.5 * 3 months 1. 72.8 2. 73.3 3. 68.3 4. 74.6 5. 76.8 6. 78.4	Only ISQ; * Baseline * 3 months	No significant difference between the six surfaces. No statistical correlation roughness surface—RFA at baseline. Statistical correlation roughness-surface at secondary stability and BIC
Do Carmo (2018) [38]	80 imp, 119 patients	Human, In vivo. RTC.	1. Double etching (Osseotite) 2. Double etching with deposition of CaP Crystal (Nanotite) 3. Sandblasted and etched (SLA) 4. Sandblasted and etched, and made hydrophilic (SLActive)	Sa; 1. 0.68 µm. 2. 0.65 µm. 3. 1.78 µm. 4. 1.75 µm.	ISQ: * Implant Placement 1. 77 ± 4.9 2. 79 ± 4.8 3. 77 ± 5.2 4. 78 ± 4.0 * day 91; 1. 79 ± 3.7 2. 81 ± 2.4 3. 82 ± 2.1 4. 82 ± 1.6 IT 1. 44.4± 6.6 2. 46.8 ± 5 3. 43.8 ± 6.5 4. 43.9 ± 6.1	IT; Implant placement. ISQ; * Implant placement. * weekly between 7 and 91 days post-surgery	IT values similar (*p* > 0.05) for all implant types. At 91 days, ISQ significantly higher than baseline for all implants (*p* < 0.001). ISQ and IT significantly correlated. No statistical correlation roughness surface—RFA
Gottlow (2012) [41]	180 implants	Animal, in vivo. 30 rabbits	1. Oxidized (Replace) 2. Hydrophilic sand-blasted and acid etched (SLActive)	Sa; 1. 1.5 µm. 2. 1.78 µm.	ISQ: * Implant placement 1. 76-80 2. 65-76 * 6 weeks 1. 84-87 2. 85-88	ISQ; * Implant placement * 10 days, 3, 6 weeks	Surfaces increased stability from placement to after 6 weeks. No statistical correlation roughness surface—RFA
Kim (2010) [42]	30	Animal, in vivo. 5 Dogs	1. Machined 2. Sandblasted large grit and acid etched 3. Anodized by oxidized electricity	Sa values; 1. 0.86 µm. 2. 1.76 µm. 3. 1.02 µm.	ISQ: * Baseline; 1. 71.33 ± 2.42 2. 71.67 ± 3.33 3. 71.83 ± 2.48 * 10 weeks 1. 70.83 ± 3.31 2. 72.83 ± 1.94 3. 72.67 ± 1.75	ISQ; * Baseline * 3, 6 and 10 weeks after surgery	ISQ significantly different among 3 groups. No statistical correlation roughness surface—RFA. May have significant effects on biological stability (3 weeks).
Kormoczi (2021) [43]	75 implants, 60 patients	Human, in vivo. Prospective.	1. alumina sandblasted and acid-etched (SA) 2. bioabsorbable apatite nano- coating (NH) 3. large-grit sandblasted and acid-etched (SLA)	Ra values; 1. 2.5–3 μm. 2. 2.5–3 μm 3. 1.42 μm	ISQ: * Baseline 1. 55.69 ( ± 15.7) 2. 59.11 ( ± 19.5) 3. 65.95 ( ± 9.8) * 6 weeks 1. 63.44 ( ± 16.7) 2. 64.10 ( ± 19.7) 3. 67.85 ( ± 9.9)	ISQ; * Baseline * Six weeks	All the ISQ values increased after six weeks. No statistical correlation roughness surface—RFA.
Qamheya (2018) [44]	15	Animal, in vivo. Sheep	1. Sandblastng and acid etching (SLA) 2. Sandblasting and thermal oxidation (SO) 3. Sandblasting, thermal oxidation, and HF acid etching (SOF)	Ra values: 1. 0.87 μm 2. 1.12 μm 3. 0.55 μm	ISQ: (SD) * Baseline: 1. 42.28 (13.4) 2. 52.39 (6.06) 3. 47.36 (6.93) * 3 weeks: 1. 61.11 (7.51) 2. 56.22 (5.76) 3. 62.56 (5.29) * 8 weeks: 1. 59.33 (11.2) 2. 60.22 (5.54) 3. 59.00 (4.74) IT: 1. 20 N/ cm 2. 18 N/cm 3. 15 N/cm	ISQ: * Baseline * 3 weeks * 8 weeks IT: * Baseline * Ba * Bas	No statistically significant correlation between any of the variables. Surface type did’t influencie osseointegration. No statistical correlation roughness surface—RFA No correlation IT-RFA.
Romero (2023) [45]	234	In vitro, calf ribs.	1. Sand blasting minimally rough surface (Tissue level) 2. Sand blasting minimally rough surface (Bone level) 3. Sand blasting rough surface (Tissue level) 4. Sand blasting rough surface (bone level) 5. Sand blasting moderately rough surface (Tissue level) 6. Sand blasting moderately rough surface (bone level)	Sa values: 1. 0.55 ± 0.01 2. 0.54 ± 0.07 3. 3.85 ± 0.18 4. 2.76 ± 0.21 5. 1.60 ± 0.22 6. 1.67 ± 0.19	ISQ: * Surgery 1. 64.1 ± 5.4 2. 70.7 ± 8.5 3. 63 ± 8.1 4. 73 ± 4.4 5. 59.6 ± 9.5 6. 72 ± 5.7 IT: 1. 25.8 ± 10.4 2. 29.4 ± 11.8 3. 28.4 ± 11.4 4. 14.6 ± 4.35 5. 29 ± 11 6. 15.2 ± 7.4	ISQ and IT: * Surgery	Rough surfaces with Sa values of 0.5 to 4 µm do not affect the primary stability. No statistical correlation roughness surface—RFA. Statistical correlation between ISQ and IT.
Souza (2019) [46]	20	Animal, in vivo. Rabbits	1. machined (control) 2. Test: Al_2_O_3_ sandblasting and acid etching	Ra values; 1. 0.46 ± 0.1 μm 2. 1.1 ± 0.16 μm	ISQ: * Baseline: 1. 48.1 ± 2.9 2. 50 ± 2 * 3 weeks; 1. 51.6 ± 2.3 2. 53.5 ± 1.9 * 6 weeks: 1. 52 ± 2 2. 54.75 ± 0.8	ISQ: * Baseline * 3 weeks * 6 weeks	Higher statistically significant ISQ values in treated group. Statistical correlation surface roughness—RFA.
Strnad (2008) [48]	24	Animal, in vivo. Beagle dogs.	1. Turned, machined (control) 2. sandblasted, acid and alcali treated. (test)	* Sa values: 1. 0.3–0.5 2. 1.1–1.3	ISQ: * Baseline: 1. 74.5 ± 2.99 2. 74 ± 2.45 No significant difference * 12 weeks 1. 73 ± 2.37 2.75 ± 2.28	ISQ: * Baseline * 1, 3, 9, 12 weeks	Test surface enhances secondary stability. No correlation RFA-BIC. No statistical correlation roughness surface—RFA
Sul (2009) [47]	60	Animal, in vivo. Rabbits	1. Oxidized Mg incorporated 2. Oxidized MgMp incorporated 3. Anodized (Ti Unite) 4. Double etching (Osseotite) 5. Sandblasted and etched (SLA) 6. TiO_2_ blasted (TiOblast)	Sa values; 1. 0.7 ± 0.2 2. 0.7 ± 0.2 3. 1.3 ± 0.1 4. 0.7 ± 0.4 5. 1.2 ± 0.2 6. 0.9 ± 0.3	ISQ * Baseline: 1. 67.9 ± 1.4 2. 66.2 ± 0.6 3. 68.6 ± 1.7 4. 67.8 ± 0.9 5. 67.9 ± 0.9 6; 68 ± 1.1 * 6 weeks: 1. 73.1 ± 2.1 2. 75.2 ± 1.8 3. 73.5 ± 2.3 4. 71.5 ± 2.5 5. 72.4 ± 2.6 6. 72.2 ± 3.1	ISQ: * Baseline * 6 weeks	Implant surface influence secundary stability. No statistical correlation roughness surface—RFA

## Data Availability

The raw data supporting the conclusions of this article will be made available by the authors on request.
